# TRPC1 channel clustering during store-operated Ca^2+^ entry in keratinocytes

**DOI:** 10.3389/fphys.2023.1141006

**Published:** 2023-03-06

**Authors:** Declan Manning, Richard Barrett-Jolley, Richard L. Evans, Caroline Dart

**Affiliations:** ^1^ Institute of Systems, Molecular and Integrative Biology, University of Liverpool, Liverpool, United Kingdom; ^2^ Institute of Life Course and Medical Sciences, University of Liverpool, Liverpool, United Kingdom; ^3^ Unilever Research & Development, Port Sunlight Laboratory, Wirral, United Kingdom

**Keywords:** orai channels, TRPC channels, keratinocytes, store-operated calcium entry, store-operated channels, skin, epidermis, differentiation

## Abstract

Skin is the largest organ in the human body with ∼95% of its surface made up of keratinocytes. These cells maintain a healthy skin barrier through regulated differentiation driven by Ca^2+^-transcriptional coupling. Many important skin conditions arise from disruption of this process although not all stages are fully understood. We know that elevated extracellular Ca^2+^ at the skin surface is detected by keratinocyte Gα_q_-coupled receptors that signal to empty endoplasmic reticulum Ca^2+^ stores. Orai channel store-operated Ca^2+^ entry (SOCE) and Ca^2+^ influx *via* “canonical” transient receptor potential (TRPC)-composed channels then activates transcription factors that drive differentiation. While STIM-mediated activation of Orai channels following store depletion is well defined, how TRPC channels are activated is less clear. Multiple modes of TRPC channel activation have been proposed, including 1) independent TRPC activation by STIM, 2) formation of Orai-TRPC-STIM complexes, and 3) the insertion of constitutively-active TRPC channels into the membrane during SOCE. To help distinguish between these models, we used high-resolution microscopy of intact keratinocyte (HaCaT) cells and immunogold transmission electron microscopy (TEM) of HaCaT plasma membrane sheets. Our data shows no evidence of significant insertion of Orai1 or TRPC subunits into the membrane during SOCE. Analysis of transmission electron microscopy data shows that during store-depletion and SOCE, Orai1 and TRPC subunits form separate membrane-localized clusters that migrate towards each other. This clustering of TRPC channel subunits in keratinocytes may support the formation of TRPC-STIM interactions at ER-plasma membrane junctions that are distinct from Orai-STIM junctions.

## 1 Introduction

Keratinocytes protect the body from the extracellular environment by forming a condensed layer of cornified epithelial tissue that covers the proliferative cell layers below. The cornified layer is produced as keratinocytes crowd towards the skin surface and differentiate in response to increased extracellular calcium [Ca^2+^]_o_ (Capone et al., 2000; Hofer and Brown, 2003; Tu et al., 2004). Elevated [Ca^2+^]_o_ is detected by keratinocytes *via* the extracellular Ca^2+^-sensing receptor (CaSR), which couples to Gα_q_ proteins to activate phospholipase C (Hofer & Brown, 2003; Tu et al., 2005). This in turns stimulates IP_3_-mediated Ca^2+^ mobilization from the endoplasmic reticulum (ER), emptying Ca^2+^ stores and triggering the opening of store-operated channels in the plasma membrane (Numaga-Tomita and Putney, 2013). Incoming Ca^2+^ then reaches the nucleus where it activates specific transcriptional factors, including the critical differentiation regulator activating protein 1 (AP-1). This form of Ca^2+^-transcriptional coupling notably upregulates the expression of many structural proteins, including involucrin and keratin, and also alters the expression of hundreds of other genes to shift the cell towards a differentiated phenotype (Rossi et al., 1998; Capone et al., 2000; Ng et al., 2000; Tu et al., 2004). A number of skin diseases including Darier’s disease, anhidrotic ectodermal dysplasia, Hailey’s disease and psoriasis arise from failures of epidermal Ca^2+^ handling (Pani and Singh, 2008; Lacruz and Feske, 2015; Dai et al., 2021), although several stages in the keratinocyte Ca^2+^-transcription pathway are not well understood.

Store-operated Ca^2+^ entry (SOCE) through Orai channels is a critical component of keratinocyte differentiation (Numaga-Tomita and Putney, 2013) and Ca^2+^ influx *via* “canonical” transient receptor potential (TRPC)-composed channels also plays an essential role (Tu et al., 2005; Fatherazi et al., 2007; Beck et al., 2008; Muller et al., 2008). Orai channels are directly activated by STIM proteins which themselves directly sense the Ca^2+^ levels within the internal stores (Emrich et al., 2022). However, there is no consensus for how TRPC channel opening is triggered by CaSR activation. Aside from activation by incoming “trigger” Ca^2+^ (Blair et al., 2009; Gross et al., 2009), some members of the TRPC family can be directly activated by diacylglycerol downstream of G_q_ protein activity (Hofmann et al., 1999; Trebak et al., 2003; Storch et al., 2017). However, there is also evidence for the activation of TRPC channels by STIM (Huang et al., 2006; Yuan et al., 2007; Alicia et al., 2008), the formation of Orai-TRPC-STIM complexes (Jardin et al., 2008; Jia et al., 2017), and the insertion of constitutively active TRPC channels into the membrane during SOCE (Cayouette et al., 2004; Cheng et al., 2011).

To distinguish between these latter three models, we used high-resolution microscopy of intact keratinocyte (HaCaT) cells to determine membrane insertion, and immunogold transmission electron microscopy of plasma membrane sheets to measure the spatial relationship between Orai1 and TRPC channel subunits at rest, and during store-depletion and SOCE.

## 2 Materials and methods

Unless specified otherwise, all reagents were purchased from Sigma-Aldrich (St Louis, MO, United States). Experiments were undertaken at room temperature (20°C–25°C) unless indicated otherwise.

### 2.1 Cell culture

The HaCaT keratinocyte cell line was gifted from David Fernig (University of Liverpool, United Kingdom) and validated by 16-loci short tandem repeat profiling at the European Collection of Authenticated Cell Cultures (ECACC). Cells were cultured under sterile conditions at 37°C, 5% CO_2_ and sub-cultured every 3–4 days. Differentiated HaCaT cells were cultured in Dulbecco’s Modified Eagle’s Medium (DMEM) supplemented with 2 mM L-Glutamine and 10% (v/v) foetal bovine serum (FBS) (Thermo Fisher Scientific Inc., Waltham, MA, United States). To maintain a basal phenotype, HaCaT cells were cultured in low-Ca^2+^ DMEM (Thermo Fisher Scientific) supplemented with 30 µM CaCl_2_ and 10% (v/v) FBS following Ca^2+^ chelation with 0.76% (w/v) Chelex-100 resin (Wilson, 2014).

### 2.2 RNA extraction, DNAse treatment and reverse-transcription

RNA was extracted using an RNeasy Mini Kit^®^ (Qiagen, Valancia, CA, United States) according to manufacturer’s instructions. RNA was treated with DNAse I to remove contaminating genomic DNA before cDNA was synthesized using SuperScript^®^ III reverse transcriptase (Invitrogen) according to manufacturer’s instructions.

### 2.3 Quantitative reverse-transcriptase (qRT-) PCR

Primers were obtained from Sigma-Aldrich or GeneGlobe (Qiagen; Supplementary Table S1). qRT-PCR reactions contained up to 200 ng cDNA, forward and reverse primers (10 nM), and PowerUp SYBR Green Mastermix 1X (Thermo Fisher Scientific) topped up to 25 µL with RNAse-free water (Thermo Fisher Scientific). Reactions were amplified with a StepOnePlus thermal cycler (Thermo Fisher Scientific). PCR reactions were initially subjected to a 95°C holding stage for 10 min followed by 48 cycles consisting of 15 s at 95°C followed by 60 s at 60°C. mRNA expression was calculated using the ΔΔCt method based on the formula RQ = 2^−ΔΔCt^ (Livak and Schmittgen, 2001). No-template control reactions all failed to amplify any genetic material and melt curve analyses indicated no amplification of off-target products in each reaction. mRNA expression was normalised to β-actin and presented as mean ΔCt values or values relative to Orai1.

### 2.4 Antibodies

The following primary antibodies were used for immunofluorescence: mouse anti-Orai1 (sc-377281, Santa Cruz Biotechnology) and rabbit anti-TRPC1 (PA5-77303, Invitrogen). The following primary antibodies were used for gold particle transmission electron microscopy (TEM): rabbit anti-Orai1 (SAB3500412, Sigma) and mouse anti-TRPC1 (sc-133076, Santa Cruz Biotechnology). Secondary antibodies for immunofluorescence were: Alexa Fluor 488-conjugated anti-mouse IgG and Alexa-fluor 647 anti-rabbit IgG (Invitrogen). Primary antibodies for TEM were directly conjugated to gold particles. Antibodies were assessed for their ability to detect the Orai1 or TRPC1 using HEK293 cells transiently expressing epitope-tagged versions of the proteins (Supplementary Figures S1, S2). We also assessed the cross-reactivity of anti-Orai1 antibodies with Orai2 and Orai3 (Supplementary Figures S1, S2).

### 2.5 Immunofluorescence

HaCaT cells were grown on poly-L-lysine-coated coverslips and fixed in 4% (w/v) paraformaldehyde (PFA), 0.2% (v/v) Triton X-100 in PBS for 10 min. Live cells were stained prior to fixation with 1X CellBrite 555 Fix in PBS (Biotium; 60 min). Fixed cells were washed with PBS and blocked in buffer containing 1% (w/v) bovine serum albumin (30 min) before incubating with primary antibody solutions in blocking buffer (1:50 dilution for 60 min). Cells were washed before addition of fluorophore-conjugated anti-IgG secondary antibodies in blocking buffer (30 min). Coverslips were mounted onto glass slides using Prolong Glass Antifade Mountant (Thermo Fisher Scientific). Images were captured through a ×63 oil-immersion objective (Zeiss) using the lattice-structured illumination microscopy (lattice-SIM) mode on an Elyra-7 super resolution microscope (Zeiss). 200 mW laser lines were used to stimulate fluorophores at 488, 561, and 642 nm wavelengths, at 2% laser intensity. Relevant excitation, filtering and grating parameters for each channel are detailed in Supplementary Table S2. Images were captured with 50 ms camera exposure time in 16-bit grey depth and z-stack images were captured every 101 nm, producing approximately 100 stacks per image.

### 2.6 Lattice-SIM image processing and analysis

Z-stack image files encompassing 2-6 cells were sectioned within ImageJ 2.3 (Rasband, W.S., ImageJ, U. S. National Institutes of Health, Bethesda, Maryland, United States, https://imagej.nih.gov/ij/, 1997–2018), removing the lower 10–20 z-planes to avoid any non-specific binding of CellBrite to the poly-L-lysine coverslip coating. Single-cell images were pre-processed using the SIM^2^ formula package for Zen Black 16.0 (Zeiss). 3-dimensional SIM^2^ processing was applied using the “Weak, Fixed” pre-set (Low input signal-to-noise ratio, 15 iterations, regularization weight 0.065) and a median filter was applied using the ‘Fast Fit’ method. Pre-processed z-stacks were exported into Imaris 9.8 (Andor) for image analysis. Binary “Surface” objects were segmented, firstly marking the plasma membrane (CellBrite 555 Fix) using smoothing (surface detail 0.0626 μm) and thresholding *via* background contrast (largest sphere diameter 1.00 μm). Total Orai1 and TRPC1 surfaces were also generated with smoothing (surface detail 0.0626 μm) and background contrast thresholding (largest sphere diameter 0.235 μm). “Split touching Objects (Region Growing)” was enabled to watershed closely aligned surfaces (seed points diameter 0.313 μm). Seed points were subjected to a median intensity filter to reduce non-specific signal. Finally, membrane-restricted Orai1 and TRPC1 surfaces were generated by strictly assessing surfaces overlapping the membrane volume (>10^–20^ μm^3^, the lower volume limit in Imaris 9.8). Surface statistics were exported into Excel 16.5 (Microsoft) to analyse volume overlap and nearest-neighbour distances.

### 2.7 Immunogold labelling and transmission electron microscopy

Plasma membrane sheets were prepared from cultured HaCaT cells grown on poly-L-lysine-coated glass coverslips as previously described (Prior et al., 2003). For details on grid preparation see Supplementary Methods. Grids were imaged using a FEI 120 kV Tecnai G 2 Spirit BioTWIN transmission electron microscope. Distances between gold particles were measured using ImageJ 2.3 and exported to Excel 16.5 (Microsoft) for nearest-neighbour distance calculation and Prism 9 (GraphPad) for analysis. Images were processed using ImageJ 2.3 and Excel 16.5 (Microsoft Corp, United States) prior to analysis and plotting in Prism 9 (GraphPad Software, Inc., San Diego, United States). Density-based spatial clustering of applications with noise (DBSCAN) was implemented in R (Hahsler, 2019). Epsilon neighbourhood and minimum cluster size were set to 50 and 5 respectively. Other parameters were defaults.

### 2.8 Statistical analysis

Statistical tests and *p* values are stated throughout.

## 3 Results

### 3.1 HaCaT keratinocytes express transcripts for Orai1, 2, 3, STIM1, 2 and TRPC1 and 4

**FIGURE 1:**
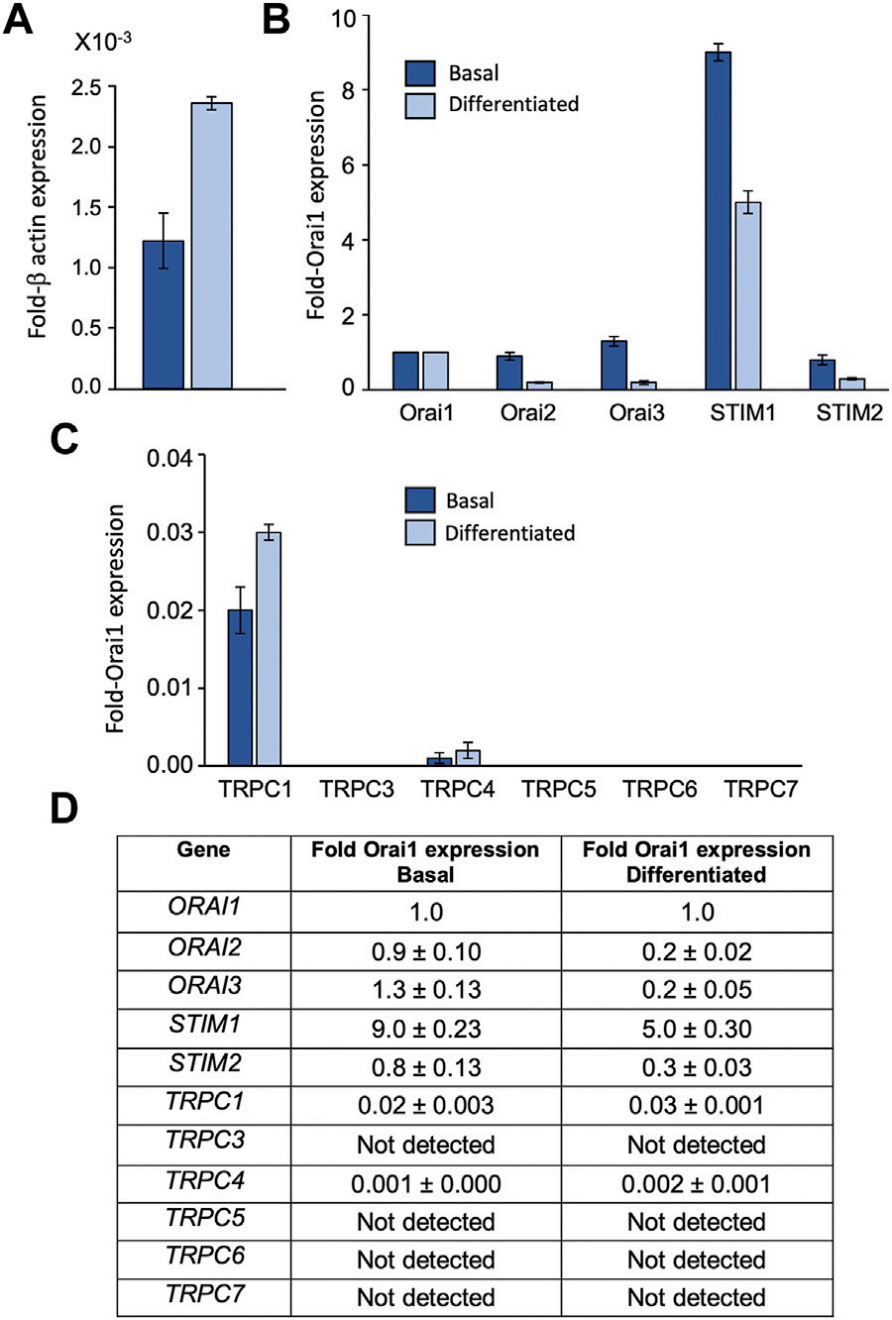
HaCaT keratinocytes express transcripts for Orai1, 2, 3, STIM1, 2 and TRPC 1, 4 HaCaT mRNA expression from basal (dark blue) and differentiated HaCaT cells (light blue) as determined by qRT-PCR. Results are presented relative to β-actin **(A)** or Orai1 expression **(B,C)**. All STIM, Orai and TRPC results are shown as mean ± SEM for *N* = 3 experimental replicates **(D)**.

To assess the relative transcript levels of TRPC, Orai and STIM isoforms in HaCaT keratinocytes we used qRT-PCR to screen for mRNA expression in basal and differentiated cells. Transcripts for Orai1, Orai 2 and Orai3 were found in both basal and differentiated HaCaTs (Figures 1A, B, D). Orai1 expression, represented as a fraction of β-actin, was found to approximately double between basal and differentiated cells (Figure 1A). Orai2 and Orai3 expression were similar to Orai1 in the basal phenotype, but both Orai2 and Orai3 expression reduced with differentiation (Figure 1B). STIM1 was the dominant STIM isoform in both HaCaT phenotypes (Figures 1B, D). STIM2 was detected at much lower levels such that the approximate STIM1:STIM2 ratio was 10:1 in the basal phenotype versus 18:1 in the differentiated phenotype. TRPC1, C3, C4, C5, C6, and C7 transcript levels were also determined (Figures 1C, D). TRPC1 was the dominant isoform and C4 was also detected. Both TRPC variants were detected at low levels relative to Orai1 and remained relatively constant between basal and differentiated phenotypes. TRPC3, C5, C6, and C7 mRNA were not detected in samples from either HaCaT phenotype.

### 3.2 Orai1 and TRPC1 channel subunits co-localize during SOCE: Lattice-SIM imaging

Keratinocyte store-operated Ca^2+^ entry is driven largely by Orai1 (Numaga-Tomita and Putney, 2013). We thus chose to focus on this isoform for high-resolution lattice-structured illumination microscopy (lattice-SIM) studies to examine Orai-TRPC co-localization and Orai/TRPC membrane insertion during SOCE. Only TRPC1 and C4 have been implicated previously in keratinocyte store-operated currents (Cai et al., 2005; Cai et al., 2006; Fatherazi et al., 2007; Beck et al., 2008). Of these two TRPC variants that we detected at transcript level, only antibodies against the dominant TRPC1 were found to be suitable for use in combination with Orai1 antibodies in immunofluorescence experiments. We therefore selected TRPC1 for this investigation. Due to the number of other members of the TRPC family, cross-reactivity of the anti-TRPC1 antibodies was not assessed. These antibodies robustly detected epitope-tagged TRPC1 in transient expression systems (Supplementary Data), but we cannot exclude the possibility that in keratinocytes these antibodies cross-react with another TRPC protein.

**FIGURE 2:**
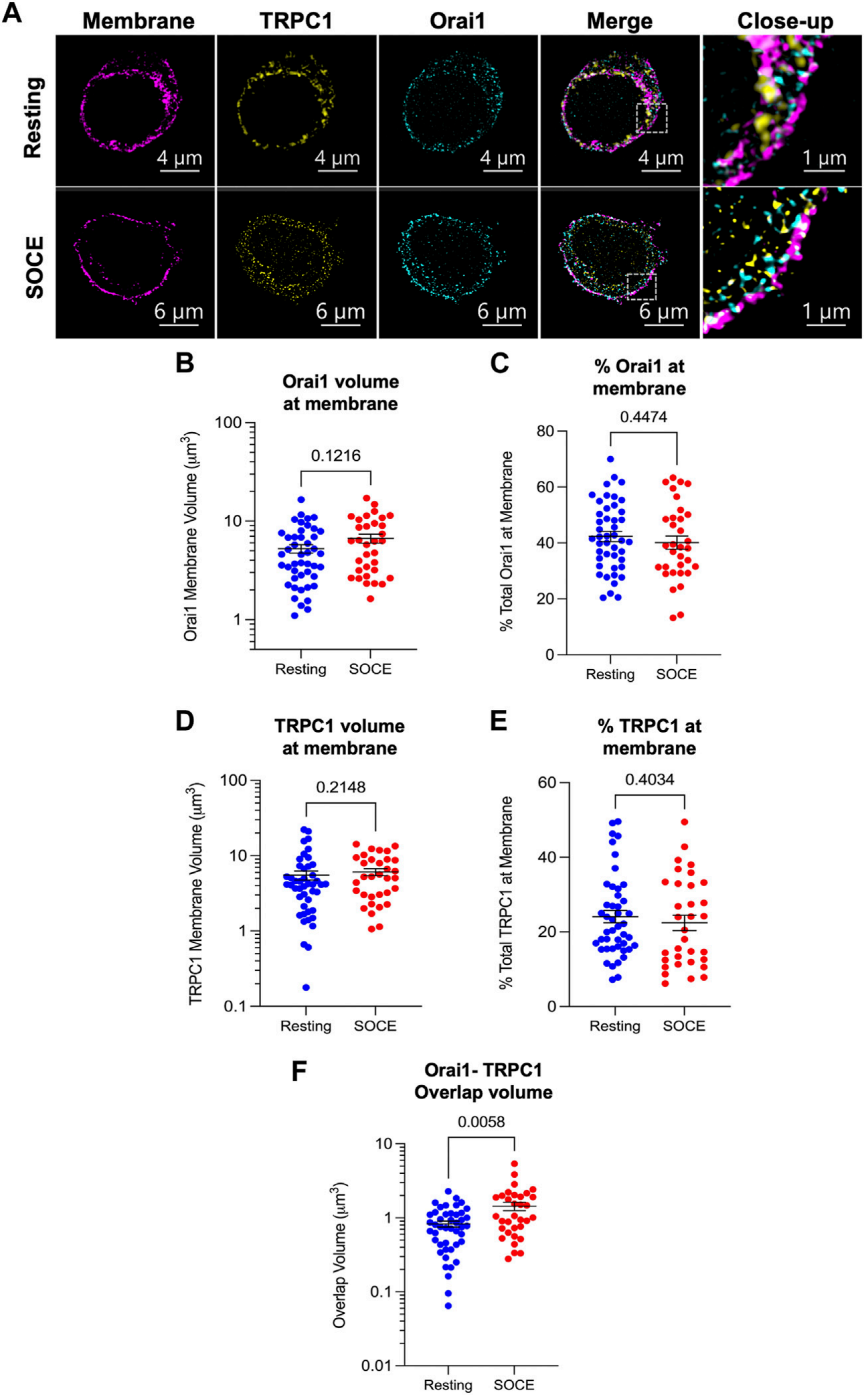
Orai1 and TRPC1 co-localize during SOCE but membrane levels of both proteins remain constant. **(A)** Representative lattice-SIM immunofluorescent images of HaCaT cells fixed in a resting state (upper panel) or following activation of store-operated Ca^2+^ entry (lower panel). Cells were stained for the plasma membrane (CellBrite 555 Fix), and with antibodies against TRPC1 and Orai1. Images show a transverse slice from a z-stack which was used to construct 3D models for co-localization analysis. Staining for TRPC1 and Orai1 was identified using the surfaces methodology in Imaris v9.8 (see Section 2). Surfaces of TRPC1/Orai1 signal were filtered to staining coincident with the plasma membrane stain and watershed to represent individual clusters of fluorescence signal. Resultant surfaces were analysed for overlap of Orai1 **(B,C)**, TRPC1 **(D,E)** and with the plasma membrane stain and for total overlapping volume of Orai1-TRPC1 **(F)** under both resting (blue) and SOCE (red) conditions.

Immunofluorescence experiments were conducted using antibodies targeted to Orai1 and TRPC1 in conjunction with plasma membrane staining (CellBrite 555 Fix; Figure 2A). Cells were treated with a SOCE activation protocol (5 min in nominally Ca^2+^-free PBS with 2 μM thapsigargin followed by 2 min in 2 mM Ca^2+^-supplemented PBS) or maintained in a resting condition (2 mM Ca^2+^-PBS alone) prior to fixation, permeabilization and staining. Plasma membrane staining was used to stratify Orai1 and TRPC1 signal incident at the plasma membrane. The resultant membrane-restricted TRPC1/Orai1 signal was used for co-localization and membrane expression analysis.

TRPC1 and Orai1 membrane expression was assessed by examining the volume of signal co-incident with the plasma membrane stain (Figures 2A–E). Membrane-incident Orai1 volume was 4.59 μm^3^ in resting cells (median, IQR 2.68–7.23 μm^3^; Figure 2B, blue) versus 6.24 μm^3^ in the SOCE condition (IQR 2.96–9.93 μm^3^; red; Mann-Whitney test, *p* = 0.1216). These values correspond with 41.5% of total Orai1 signal in resting cells (IQR 32.9%–51.8%; Figure 2C, blue) versus 38.5% of total Orai1 signal in the SOCE condition (IQR 30.3%–49.6%; red; *p* = 0.4474). Membrane-incident TRPC1 volume was 4.16 μm^3^ in resting cells (median, IQR 2.01–6.98 μm^3^; Figure 2D, blue) versus 5.22 μm^3^ in the SOCE condition (IQR 2.93–8.91 μm^3^; red; *p* = 0.2148). As a percentage of the total TRPC1 signal detected in each cell, these values correspond with 21.4% of total TRPC1 in resting cells (IQR 15.8%–30.7%; Figure 2E, blue) versus 20.5% of total TRPC1 in the SOCE condition (IQR 12.2%–33.1%; red; *p* = 0.4034). This suggests that neither Orai1 nor TRPC1 membrane expression vary significantly during HaCaT SOCE.

To assess the spatial relationship of the proteins in the membrane and how this changes during store depletion and Ca^2+^ entry we examined their co-localization. The total volume of coincident Orai1 and TRPC1 signal in the resting condition was 0.75 μm^3^ (median; IQR 0.43–1.13 μm^3^; *N* = 45 cells; Figure 2F, blue), compared with a significantly increased coincident volume in the SOCE condition of 1.05 μm^3^ (IQR 0.68–1.96 μm^3^; *N* = 33 cells; red; Mann-Whitney test, *p* = 0.0058). This suggests that a greater number of Orai1 and TRPC1 molecules are co-localized during SOCE compared with baseline.

### 3.3 Orai1 and TRPC1 channel populations cluster and co-localize during store depletion and SOCE: Gold particle immunolabelling TEM

**FIGURE 3:**
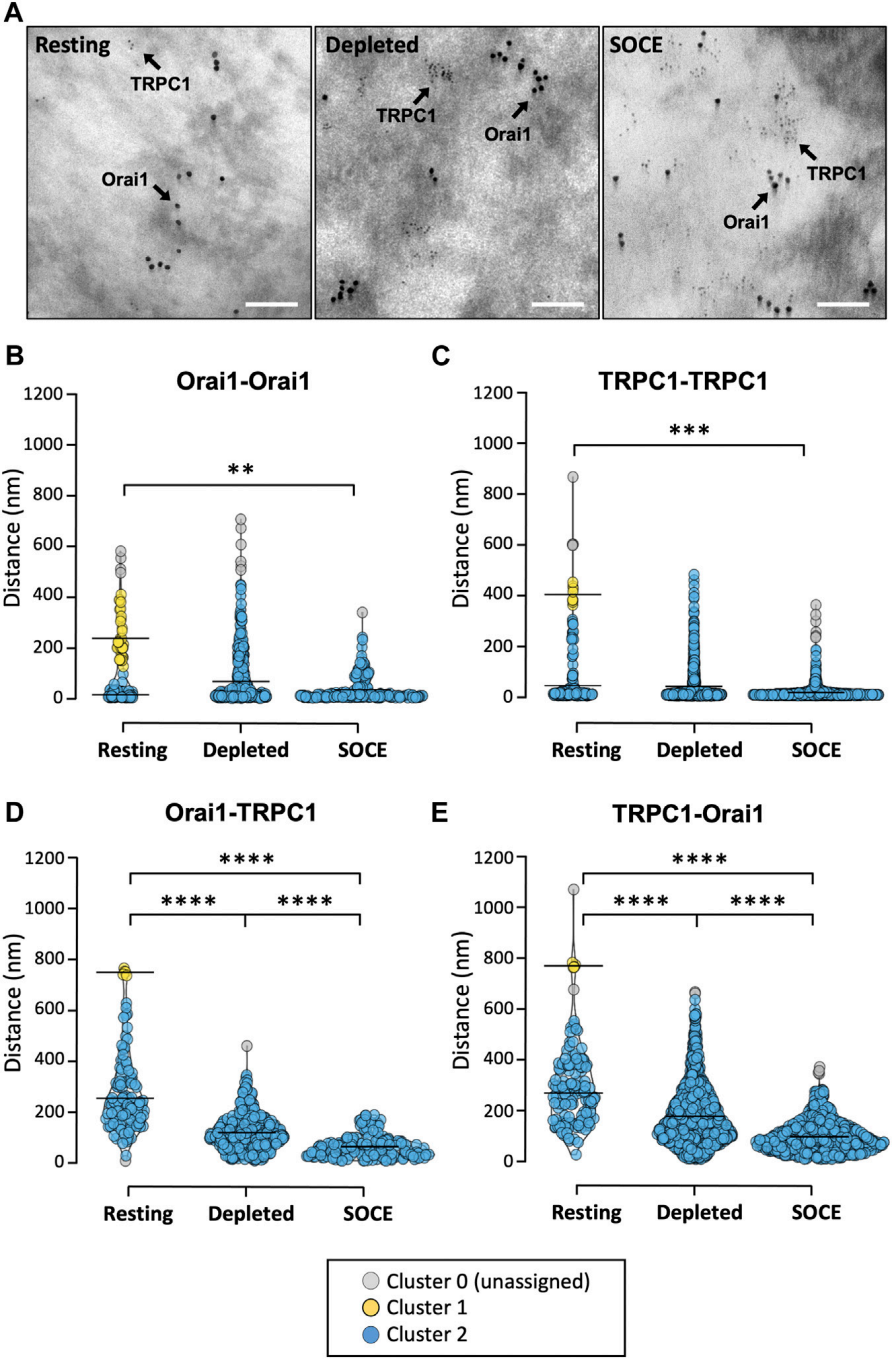
TRPC1 and Orai1 channel populations cluster and co-localize under Ca^2+^ store depletion and SOCE conditions. **(A)** Representative transmission electron micrographs of HaCaT cell membrane rip-offs (*N* = 3 biological replicates per condition) immunolabelled with 10 nm and 3 nm diameter gold nanoparticles targeted to Orai1 and TRPC1, respectively (scale bar 100 nm). **(B)** Distances between gold particles were measured to calculate nearest-neighbour distances and the DBSCAN technique used to assess whether these Euclidean distances were clustered and whether identified cluster configurations altered with treatment. Clustering was quantified for four different distance combinations [Orai1-Orai1**(B)**; TRPC1-TRPC1 **(C)**; Orai1-TRPC1 **(D)**; TRPC1-Orai1 **(E)**] under three conditions each (at rest, during store depletion, and during SOCE). For all conditions two clusters [cluster 1 (yellow) and cluster 2 (blue)] were detected at rest. Cluster 0 (grey) denotes particles not attributed to any cluster. Only one cluster (blue) was detected in all conditions following store depletion and SOCE. The mean distance between particles in each identified cluster is indicated by the horizontal line. Significance levels refer to the median distance between all particles within the population for a given condition (Kruskal-Wallis with Dunn’s multiple comparisons; **p = 0.006; ***p = 0.0009; ****p < 0.0001; see text for details).

We sought to confirm this SOCE-induced co-localization with the higher resolution of electron microscopy. HaCaT cells were treated prior to fixation and preparation for imaging. Treatments included either: 1) 7 min in 2 mM Ca^2+^-supplemented PBS (resting); 2) 7 min in nominally Ca^2+^-free PBS with 2 μM thapsigargin (depleted); or 3) 5 min in Ca^2+^-free PBS with thapsigargin followed by 2 min in 2 mM Ca^2+^-PBS (SOCE). After treatment and fixation, basal HaCaT plasma membranes were ripped off exposing the inner membrane leaflet for immunolabelling with primary antibodies targeted to cytoplasmic portions of Orai1 and TRPC1 proteins. Primary antibodies were directly conjugated with gold nanoparticles (Orai1:10 nm diameter gold and TRPC1: 3 nm diameter gold) to distinguish between these proteins (Figure 3A). This procedure was carried out three times per condition (*N* = 3 biological replicates), with each condition totalling between 117–365 Orai1-associated particles and 156–1107 TRPC1-associated particles. Distances between gold particles were measured to calculate nearest-neighbour distances. We investigated whether these Euclidean distances were clustered, and whether such cluster configurations altered with treatment, using the density-based spatial clustering of applications with noise (DBSCAN) technique (Hahsler, 2019). Clustering was quantified for four different distance combinations (Orai1-Orai1; TRPC1-TRPC1; Orai1-TRPC1; TRPC1-Orai1) under three conditions each (at rest, following store depletion, and during SOCE).

For each distance combination, clustering analysis detected two clusters at rest and only one cluster following store depletion and SOCE (Figures 3B–E). Note that the two “clusters” at rest simply denote that some particles are “close” and others are “further away,” perhaps randomly distributed throughout the membrane. The loss of the “further away” population following treatment for each distance combination reflects that the particles move closer together. This suggests that under these conditions, Orai1 and TRPC1 subunits form separate membrane-localized clusters and that these clusters migrate towards each other.

For Orai1 channels at rest, the mean distance between Orai1 particles was 244.5 ± 80.0 nm in cluster 1 (mean ± SD; *n* = 35; Figure 3B, yellow) and 21.9 ± 14.9 nm for cluster 2 (*n* = 78; Figure 3B, blue). Following store depletion, only one cluster could be detected centred at 74.7 ± 93.8 nm (*n* = 357; Figure 3B, blue), and during SOCE only one cluster was identified centred at 42.1 ± 46.7 nm (*n* = 174; Figure 3B, blue). A similar pattern was seen for TRPC1-TRPC1 distances (Figure 3C). At rest, the mean distance between TRPC1 particles was 404.9 ± 32.9 nm in cluster 1 (*n* = 8; Figure 3C, yellow) and 45.5 ± 76.2 nm for cluster 2 (*n* = 144; Figure 3C, blue). One cluster was detected following store depletion centred at 41.8 ± 66.3 nm (*n* = 1,107, blue) and, again, only one cluster was detected during SOCE centred at 17.6 ± 19.1 nm (*n* = 823, blue). We also analysed the distance between Orai1-TRPC1 particles and, conversely, TRPC1-Orai1 under the different conditions. At rest, the mean distance between Orai1-TRPC1 was 749.6 ± 10.9 nm in cluster 1 (*n* = 5; Figure 3D, yellow) and 259.1 ± 125.0 in cluster 2 (*n* = 111; Figure 3D, blue). This was found to be similar for TRPC1-Orai1 distances at rest where cluster centres were at 776.3 ± 8.1 nm and 275.9 ± 117.0 nm for cluster 1 (*n* = 5; Figure 3E, yellow) and cluster 2 (*n* = 149; Figure 3E, blue), respectively. Here, again, the two clusters coalesced into one cluster following treatment. Following store depletion, the mean distance between Orai1-TRPC1 particles was 115.1 ± 68.5 nm (*n* = 362; Figure 3D, blue), similar to the mean distance between TRPC1-Orai1 (177.0 ± 112.6 nm, *n* = 1,105; Figure 3E, blue). During SOCE, the one identified Orai-TRPC cluster centred at 64.5 ± 43.3 nm (*n* = 175; Figure 3D, blue), and the single TRPC-Orai cluster centred at 96.4 ± 57.9 nm (*n* = 825; Figure 3E, blue).

Independent from cluster analysis, we also looked at the median distance between all particles within the population for a given condition (Supplementary Figure S3). The distance between a given labelled Orai1 particle and its nearest Orai1 neighbour was found to be significantly lower during SOCE (median 19 nm, IQR 14–52 nm) compared to resting (median 24 nm; IQR 15–206 nm; Kruskal-Wallis with Dunn’s multiple comparisons, *p* = 0.0056). Similarly, TRPC1 nearest-neighbour distances were significantly lower in the SOCE condition (median 11 nm, IQR 8–18 nm) compared to at rest (14 nm, IQR 8–54 nm; *p* = 0.006).

Orai1-TRPC1 median nearest-neighbour distances were 236 nm (IQR 163–334 nm) at rest compared to 106 nm in the depleted condition (IQR 67–154 nm) and 57 nm during SOCE (IQR 31–85 nm). A Kruskal-Wallis test with Dunn’s multiple comparisons demonstrated a statistically significant difference between all three distributions (*p* < 0.0001). Conversely, TRPC1-Orai median nearest-neighbour distances were 274 nm (IQR 169–386 nm), versus 150 nm in the depleted condition (IQR 97–236 nm) and 83 nm in the SOCE condition (54–128 nm; blue). Similar to the above, a Kruskal-Wallis test with Dunn’s multiple comparisons demonstrated a statistically significant difference between the distributions in all three conditions (*p* < 0.0001).

## 4 Discussion

Ca^2+^ influx *via* TRPC-composed channels plays a crucial role in the Ca^2+^-transcriptional coupling that underlies keratinocyte differentiation (Tu et al., 2005; Fatherazi et al., 2007; Beck et al., 2008; Muller et al., 2008). Here we investigate the spatial relationship between these channels and Orai1 in the HaCaT cell membrane following store depletion and during SOCE. Our data support the idea that under these conditions separate populations TRPC channels coalesce to form larger membrane-localized clusters that migrate towards clustered Orai channels.

To assess the spatial relationship of Orai and TRPC channels at the membrane under resting and SOCE conditions, high-resolution imaging was undertaken. Immunofluorescence experiments with cells fixed in resting and SOCE conditions suggest that plasma membrane Orai1 and TRPC1 co-localize during SOCE, as assessed by the overlap of respective fluorescence signal at the plasma membrane. Plasma membrane levels of Orai1 and TRPC1 were both found to be consistent between resting and SOCE conditions. These results are in contrast to findings that Orai1 Ca^2+^ entry recruits TRPC1 to the plasma membrane (Cayouette et al., 2004; Cheng et al., 2011) and findings that Orai1 is recruited to the membrane during ER Ca^2+^ store depletion (Woodard et al., 2008). We determined plasma membrane-restricted Orai and TRPC based on the staining of a well-characterized dye, which covalently labels the surface of live cells. Cells were stained prior to fixation and permeabilization to avoid dye internalization as much as possible. We cannot exclude the staining of some intracellular membranes however, and a proportion of the membrane-restricted TRPC1 or Orai1 signal may originate from intracellular trafficking vesicles close to the plasma membrane or the membrane of the junctional ER, (approximately 20 nm distance from the plasma membrane).

To look at the plasma membrane in isolation we turned to the higher-resolution membrane rip-off technique in conjunction with immuno-gold labelling and TEM. TEM carries a resolution limit closer to 0.1 nm (Franken et al., 2020) and so is expected to give enhanced detail of Orai1-TRPC1 localisation at the plasma membrane. Likely reflecting the relatively low abundance of these proteins, the staining density of Orai1/TRPC1-conjugated gold particles did not meet the minimum requirement for Ripley’s K Function analysis of clustering or co-localization (Prior et al., 2003), so nearest-neighbour distances were calculated. These data confirm numerous studies that show Orai channel clustering during SOCE (Xu et al., 2006; Park et al., 2009). Unbiased cluster analysis detected two distinct clusters for Orai at rest and only one cluster following store depletion. The lost cluster represents the “further away” population, suggesting that Orai particles begin to move closer together during store depletion. When analyzing Orai1 nearest-neighbor distances, the change in median distance between rest and store-depleted conditions did not reach significance. One possible explanation for this is that our study looks at endogenous Orai channels as opposed to over–expression systems which may exaggerate clustering effects. Since we could not directly assess Orai-STIM interactions in these experiments, another possibility is that the detected Orai clusters are not active clusters induced by STIM. Our results also demonstrate separate clustering of TRPC1 channel subunits, and a general migration of Orai1 clusters and TRPC1 clusters towards each other under these conditions. It is tempting to speculate that this may be evidence for TRPC1-STIM interactions at ER-plasma membrane junctions that are distinct from Orai-STIM junctions, although we have no direct evidence for this. This has been indicated previously in pancreatic acinar secretory epithelial cells (Hong et al., 2011) and also in HEK293 overexpression experiments (DeHaven et al., 2009), with specialized STIM-TRPC junctions suggested to exist independent of other STIM-Orai or STIM-Orai-TRPC junctions.

## Data Availability

Original datasets are publicly available. This data can be found here: http://cci02.liv.ac.uk/gallery/show_group/1204/ http://figshare.com/articles/dataset/Data/20407206.
