# Supplementary material for: TRPC1 channel clustering during store-operated Ca2+ entry in keratinocytes
Source: Front Physiol. 2023 Mar 6;14:1141006. doi: 10.3389/fphys.2023.1141006 (PMC10025536; doi:10.3389/fphys.2023.1141006)
Supplement: Supplementary file 1 [file DataSheet1.PDF]

Supplementary Material

TRPC1 channel clustering during store-operated Ca^{2+} entry in keratinocytes

Declan Manning, Richard Barrett-Jolley Richard L Evans, Caroline Dart*

*** Correspondence:** c.dart@liverpool.ac.uk

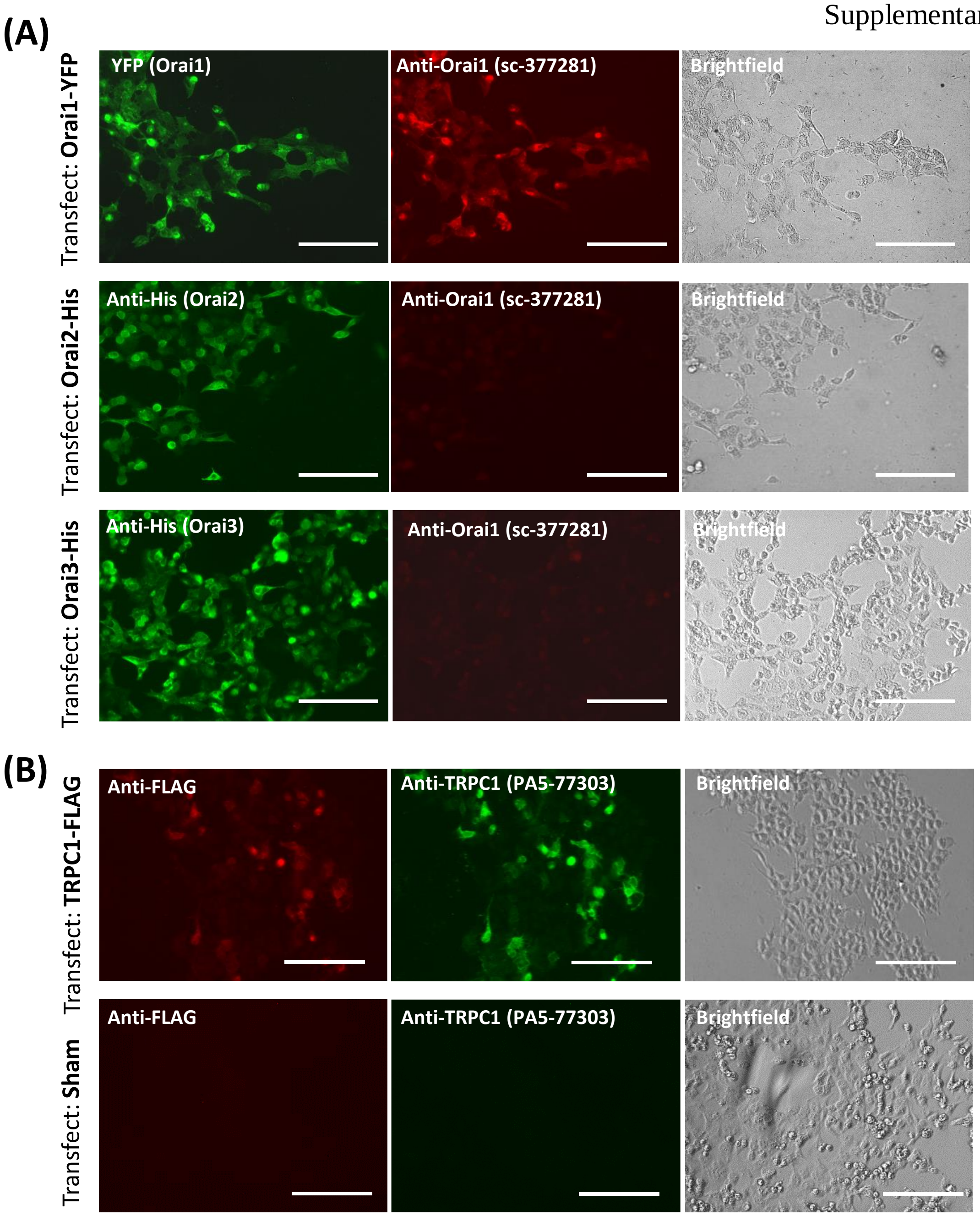

Supplementary Figure 1: Evaluation of antibodies used in immunofluorescence studies

Primary antibodies used for immunofluorescence studies were: mouse anti-Orai1 (sc-377281, Santa Cruz Biotechnology) and rabbit anti-TRPC1 (PA5-77303, Invitrogen). These antibodies were evaluated for their ability to robustly detect Orai1 (A) or TRPC1 (B) using HEK293 cells transiently expressing epitope-tagged versions of the proteins. We also assessed the cross-reactivity of anti-Orai1 antibodies with Orai2 and Orai3 (A). Due to the number of other members of the TRPC family, cross-reactivity of the anti-TRPC1 antibody was not assessed. HEK293 cells were transiently transfected with Orai1-YFP (A, upper panel); Orai2-His (A, middle panel), Orai3-His (A, lower panel), or TRPC1-FLAG (B) prior to fixation and immunostaining. Sham-transfected (no DNA) cells are shown in B. Orai1-YFP, Orai2-His and Orai3-His were gifts from Anjana Rao (Addgene plasmid #19756, #16369, #16370) and TRPC1-FLAG was a gift from Craig Montell (Addgene plasmid #24408). Orai1-YFP transfected cells were stained with mouse anti-Orai1 (sc-377281) alone. Orai2-His and Orai3-His transfected cells were co-stained with mouse anti-Orai1 (sc-377281) and rabbit anti-His (12698, Cell Signaling Technology). TRPC1-FLAG transfected cells were co-stained with rabbit anti-TRPC1 (PA5-77303) and mouse anti-FLAG (F1804, Sigma-Aldrich). Secondary antibodies were Alexa Fluor 488-conjugated anti-rabbit and Alexa-fluor 647 anti-mouse (both Invitrogen). Cells were imaged using epifluorescence microscopy (EVOS, Thermo-Fisher, UK).

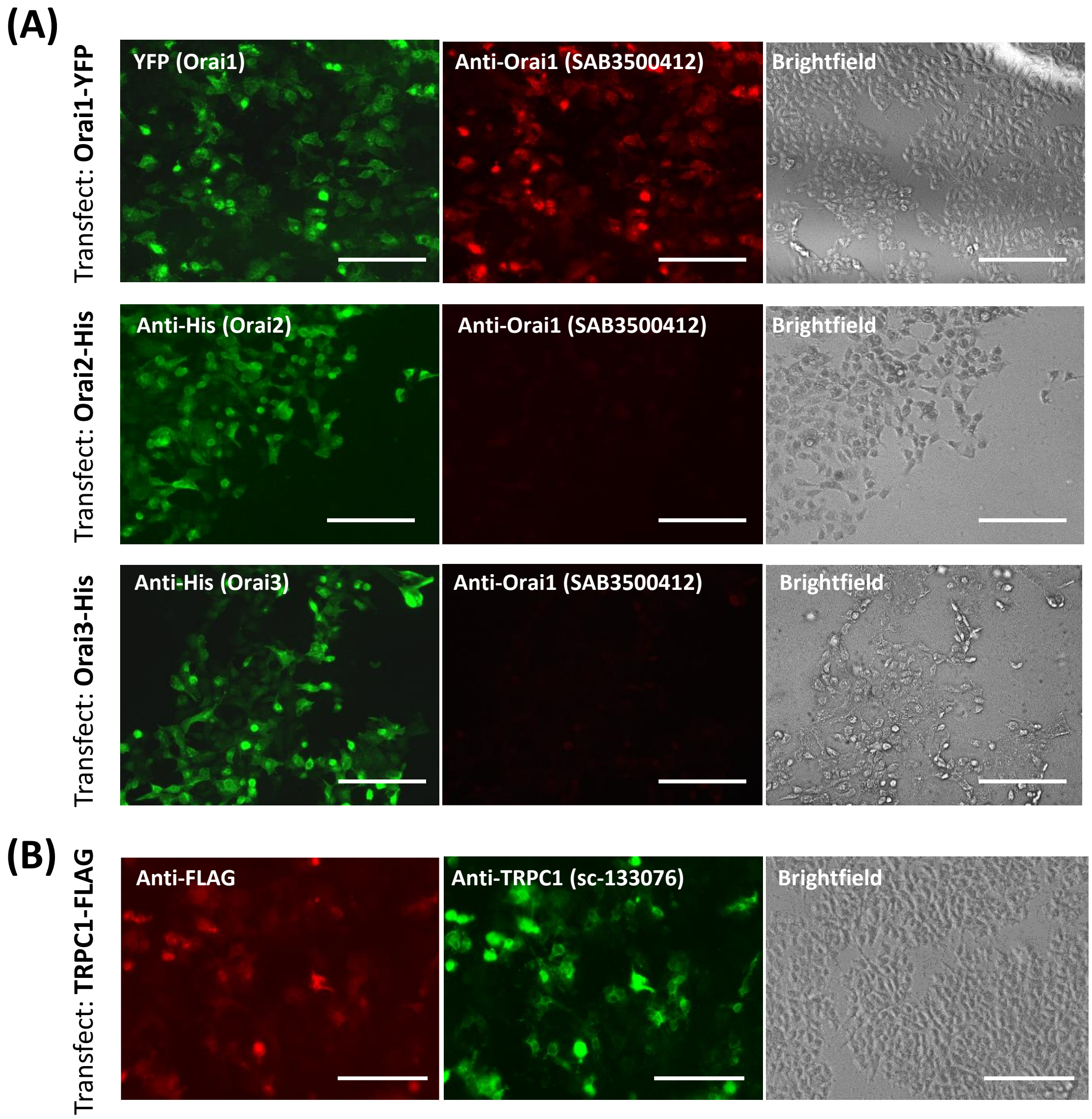

Supplementary Figure 2. Evaluation of antibodies used in immunogold labelling

Primary antibodies used for immunogold labelling were: rabbit anti-Orai1 (SAB3500412, Sigma) and mouse anti-TRPC1 (sc-133076, Santa Cruz Biotechnology). These antibodies were evaluated for their ability to detect Orai1 (A) or TRPC1 (B) using HEK293 cells transiently expressing epitope-tagged versions of the proteins. We also assessed the cross-reactivity of anti-Orai1 antibodies with Orai2 and Orai3 (A). Due to the number of other members of the TRPC family, cross-reactivity of the anti-TRPC1 antibody was not assessed. HEK293 cells were transiently transfected with Orai1-YFP (A, upper panel); Orai2-His (A, middle panel), Orai3-His (A, lower panel), or TRPC1-FLAG (B) prior to fixation and immunostaining. Orai1-YFP transfected cells were stained with rabbit anti-Orai1 (SAB3500412) alone. Orai2-His and Orai3-His transfected cells were co-stained with rabbit anti-Orai1 (SAB3500412) and mouse anti-His (MA1-135, Invitrogen). TRPC1-FLAG transfected cells were co-stained with mouse anti-TRPC1 (sc-133076) and rabbit anti-FLAG (14793, Cell Signaling Technology). Secondary antibodies were Alexa Fluor 488-conjugated anti-mouse and Alexa-fluor 647 anti-rabbit (both Invitrogen). Cells were imaged using epifluorescence microscopy (EVOS, Thermo-Fisher, UK).

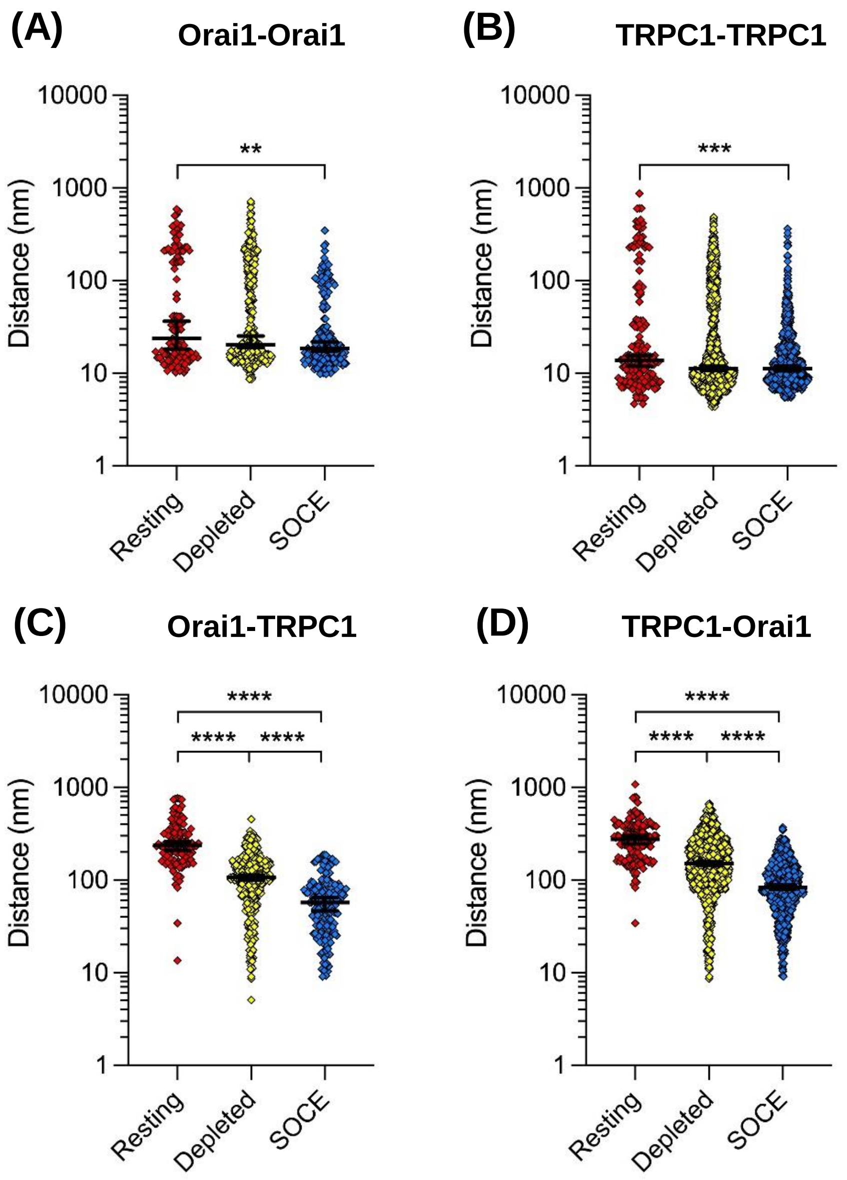

Supplementary Figure 3: Analysis of median distances between particles shows Orai and TRPC co-localize during storage depletion and SOCE.

Data shown in Figure 3 replotted using log scale on y-axis. Analysis shows that distances from a given particle to its nearest neighbour under resting conditions (red) through to ER Ca^{2+} store depletion (green) and SOCE (blue). Median value of population and IQR indicated by horizontal lines. **A.** Transition from resting to SOCE condition reduced Orai1 nearest-neighbour distances significantly (Kruskal-Wallis with Dunn's multiple comparisons, P=0.006). **B.** Distances from TRPC1 to its nearest TRPC1 neighbour under resting conditions, store depletion and SOCE. Transition from resting to SOCE condition reduced TRPC1 nearest-neighbour distances significantly (Kruskal-Wallis with Dunn's multiple comparisons, P=0.0009). Distances from Orai1 to the nearest TRPC1 particle (**C**) and TRPC1 to the nearest Orai1 particle (**D**) decreased from resting conditions (red) through to store depletion (green) and SOCE (blue). Transition from resting to depleted and then to SOCE state reduced all indicated distances significantly (Kruskal-Wallis with Dunn's multiple comparisons, all P values <0.0001).

Supplementary Methods:

Immunogold labelling & transmission electron microscopy

Grids were fixed (4% (w/v) PFA, 0.1% (w/v) glutaraldehyde in 1x KOAc Buffer: (HEPES 25 mM, potassium acetate 115 mM, $MgCl_2$ 5 mM, pH 7.40 in PBS)) and washed with 25 mM glycine in 1x KOAc buffer to quench free aldehyde groups. Blocking solution (0.2% (w/v) fish skin gelatine, 0.2% (w/v) BSA in 1x KOAc buffer) was applied for 10-15 minutes. Gold-conjugated primary antibodies diluted in blocking solution were added for 30-45 minutes. Labelled grids were washed with blocking solution as before and then washed in deionised water. Grids were stained by incubation on ice for 10 minutes in a mixture of nine parts 2% (w/v) methyl cellulose to one part 3% (w/v) uranyl acetate.

Supplementary tables:

Supplementary Table 1: Primers used in quantitative reverse-transcriptase (qRT-) PCR. Primers were either designed in-house (Orai1-3, STIM1-2) or acquired from GeneGlobe (Qiagen; TRPC1-7, neuronal ion channel array).

Target	Primer Sequence		GeneGlobe ID
	Forward	Reverse	
Orai1	GCCCTTCGGCCTGATCTTTAT	TGGAACTGTCGGTCAGTCTTAT	N/A
Orai2	TGGCGGAAGCTCTACCTGAG	CGGGTACTGGTACTGCGTC	
Orai3	TGGGTCAAGTTTGTGCCCATT	AGCTGGACTAAGGGAGGTAGC	
STIM1	TGGGATCTCAGAGGGATTTG	CATTGGAAGTCATGGCATTG	
STIM2	CCAGGGCTTTCACTGTGATT	CCTCGGCTTAAGGTTGTGAA	
TRPC1	N/A – RT^2 qPCR Primer Assay		PPH15081A
TRPC3			PPH12808G
TRPC4			PPH15312B
TRPC5			PPH14971A
TRPC6			PPH13135A
TRPC7			PPH15892A

Supplementary Table 2: Lattice-SIM imaging parameters. Fluorophores was excited with laser lines at the listed wavelengths. The resultant fluorescence was filtered with band-pass and low-pass filters and passed through diffraction grating on a specified period for each channel.

Fluorophore	Excitation	Filters		Grating Period
		Band-Pass	Low-Pass	
Alexa-Fluor 488	488 nm	495-500 nm	655 nm	27.5 µm
CellBrite Fix 555	561 nm	420-480 nm, 570-640 nm	740 nm	32 µm
Alexa-Fluor 647	642 nm	495-550 nm	655 nm	36.5 µm
